# Comparison of the Biochemical Composition and Nutritional Quality Between Diploid and Triploid Hong Kong Oysters, *Crassostrea hongkongensis*

**DOI:** 10.3389/fphys.2018.01674

**Published:** 2018-11-26

**Authors:** Yanping Qin, Yuehuan Zhang, Haitao Ma, Xiangwei Wu, Shu Xiao, Jun Li, Riguan Mo, Ziniu Yu

**Affiliations:** ^1^Key Laboratory of Tropical Marine Bio-Resources and Ecology, Guangdong Provincial Key Laboratory of Applied Marine Biology, South China Sea Institute of Oceanology, Chinese Academy of Sciences, Guangzhou, China; ^2^South China Sea Bio-Resource Exploitation and Utilization Collaborative Innovation Center, Guangzhou, China; ^3^University of Chinese Academy of Sciences, Beijing, China

**Keywords:** *Crassostrea hongkongensis*, diploid, triploid, biochemical compositions, nutritional quality, metabolic pathways

## Abstract

This study is the first systematic comparison of the biochemical composition and nutritional quality between diploid and triploid Hong Kong oysters, *Crassostrea hongkongensis*. Results showed that in the reproductive season, the glycogen content in five tissues (gill, mantle, adductor muscle, labial palps and gonad) was significantly higher (*P* < 0.05) in triploids than in diploids, with odds ratios (ORs) of 96.26, 60.17, 72.59, 53.56, and 128.52%, respectively. In the non-reproductive phase, significant differences in glycogen content (*P* < 0.05) between diploid and triploid oysters existed only in gill and gonad. In both diploid and triploid Hong Kong oysters, quantitative real-time PCR analysis of the glycogen synthesis gene (*Ch*GS) and glycogen phosphorylase gene (*Ch*GP) showed that the gene expression patterns matched the pattern of variation in glycogen content. Moreover, in both the reproductive and the non-reproductive phases, triploid Hong Kong oysters had a well balance of essential amino acids and were thus a well source of high-quality protein. Surprisingly, in both phases, significantly higher (*P* < 0.05) percentages of four essential fatty acids (α-linolenic acid, linoleic acid, eicosapentaenoic acid, and docosahexaenoic acid) were observed in triploids than in diploids. Additionally, the ratio of n-3/n-6 polyunsaturated fatty acids (PUFAs) was much higher in triploids than that in diploids. Variations in Biochemical composition were consistent with the relative expression of the citrate synthase gene (*Ch*CS) and the α-ketoglutarate dehydrogenase gene (*Ch*KD), which are key enzyme genes of the tricarboxylic acid cycle. Overall, the triploid Hong Kong oyster has a better nutritional value and taste than the diploid in terms of glycogen content, protein quality and fatty acid content.

## Introduction

Due to their rapid growth and maintaining good taste in the reproductive phase, many triploid bivalves have entered into commercial farming, including oysters (Allen and Downing, 1986; Houcke et al., 2016), scallops (Racotta et al., 2008), clams (Utting and Child, 1994; Shpigel and Spencer, 1996), and mussels (Zwaan and Zandee, 1972; Dare and Edwards, 1975). However, no solid comparative data is available on the differences in the biochemical compositions and nutritional qualities between diploid and triploid Hong Kong oysters, which are very important for promoting the application of triploid Hong Kong oysters in the industry. *Crassostrea hongkongensis* (Hong Kong oyster) is economically important and widely cultivated in southern China. However, like many other oyster species, diploid Hong Kong oysters have an inferior taste and low meat quality during the reproductive season (from May to August) each year, which can be attributed to gametogenesis (Lam and Morton, 2003; Bacca et al., 2005; Wang et al., 2015; Zhang et al., 2017). One way of solving these problems is to introduce the use of triploid oysters into the aquaculture industry (Stanley et al., 1981; Ren et al., 2003; Qin et al., 2017). Gametogenesis in triploids is retarded in the reproductive season, since triploid oysters have poorly developed gonads compared to diploids. According to previous studies on diploid *Tapes philippinarum* (Shpigel and Spencer, 1996), *Crassostrea gigas* (Soudant et al., 1999; Dridi et al., 2007; Pogoda et al., 2013), *Ostrea edulis* (Houcke et al., 2016), and *Crassostrea virginica* (Zeng et al., 2015), large changes in biochemical composition occur in response to gonad development.

Glycogen is the main molecular contributor to flavor quality in bivalves (Berthelin et al., 2000b; Racotta et al., 2008; Zhang et al., 2015). Glycogen content decreased is strongly linked with gonad development and gametogenesis in *C. gigas* (Berthelin et al., 2000b; Zeng et al., 2015). Various studies have confirmed glycogen synthesis and glycogen degradation can be altered by changes of the glycogen synthesis gene (*Ch*GS) and glycogen phosphorylase gene (*Ch*GP) (Greiwe et al., 1999; Bacca et al., 2005; Dridi et al., 2007). *Ch*GS and *Ch*GP are the key enzyme genes of glycogen synthesis and degradation, respectively, in *C. hongkongensis*. Bacca et al. (2005) confirmed that the expression level of glycogen synthase changed in a pattern consistent with seasonal variations in glycogen content in *C. gigas*, and the same phenomenon was also observed in *C. angulate* (Zeng et al., 2013).

Protein content play a important role in determing the nutritional value of food, and has a large effect on the market value (Manninen et al., 2018). At the same time, the essential amino acid composition is one of the most important factors determining the nutritional qualities of protein. Amino acid score is a good method of appraising protein quality by comparing a test amino acid content with that of the reference of the amino acid (Chen et al., 2007). When the amino acid score is calculated, it is compared to the amino acid requirements of young children. Amino acid scores are widely used for evaluating the nutritional quality of protein (Celik et al., 2004; Iqbal et al., 2006; Lv et al., 2018). In addition, it has been shown that amino acid composition is closely linked to gonad development in *C. gigas* (Kong et al., 2001; Dridi et al., 2007) and *Mytilus edulis* (Su et al., 1998).

Four essential fatty acids, namely α-linolenic acid (ALA, 18:3n-3), linoleic acid (LA, 18:2n-6), eicosapentaenoic acid (EPA, 20:5n-3), and docosahexaenoic acid (DHA, 22:6n-3) are very important for human health (Dyerberg, 1986; Coetzee and Hoffman, 2002). The nutritional quality of food is to a great extent associated with the ratio of n-3/n-6 polyunsaturated fatty acids (PUFAs). Several sources of information confirm that a higher ratio of n-3/n-6 PUFAs is desirable to reduce the incidence of many diseases (such as cardiovascular disease, asthma, breast cancer) in modern societies, especially in developed countries (Coetzee and Hoffman, 2002; Simopoulos, 2008). However, many peoples' diets are deficient in n-3 PUFAs and have excessive amounts of n-6 PUFAs (Park et al., 1994). Seafood is well known as a rich source of n-3 PUFAs, and some types of seafood have a high ratio of n-3/n-6 PUFAs (Soudant et al., 1999; Skonberg and Perkins, 2002; Celik et al., 2004; Chen et al., 2007). According to Dridi et al. (2007), PUFAs were always the dominant fatty acids in *C. gigas* throughout the year, and the ratio of n-3/n-6 PUFAs ranged from 2.45 to 3.15, indicating that *C. gigas* was a relatively nutritious food. Many researchers have found a relationship between variations in the ratio of n-3/n-6 PUFAs and gonad development; Dridi et al. (2007) confirmed that the maximum docosahexaenoic acid levels corresponded with oocyte maturation in *C. gigas*. Ojea et al. (2004) found that neutral and polar lipids increased during the period of maximum ripeness and decreased during spawning of *Ruditapes decussatus*, while Orban et al. (2002) reported high levels of n-3 polyunsaturated fatty acids during spawning of *Mytilus galloprovincialis*. However, as yet there is no study on the relationship between fatty acid composition and gonad development in *C. hongkongensis*. Our study aims to compare and assess the performance of diploid and triploid oysters during the reproductive and non-reproductive phases, from a biochemical and nutritional point of view. In addition, by illustrating the relationships between variations in biochemical composition and the gonad development of Hong Kong oysters, this study will provide important information for the application of triploid Hong Kong oysters in the aquaculture industry.

## Materials and methods

### Sample collection and ploidy determination

Diploid and triploid Hong Kong oysters for use in this study were collected from Beihai, Guangxi Province, China. Triploid Hong Kong oysters were induced by blocking the release of the second polar body (PB2) in fertilized eggs using 0.5 mg.L^−1^ Cytochalasin B in this research (Wang et al., 2002; Qin et al., 2017). Both diploid and triploid oysters used in this study were descendants from the same base population and were cultured under the same environmental conditions. Large numbers of triploid and diploid oysters were collected in December 2016 (non-reproductive phase) and July 2017 (reproductive phase). The oysters collected in July 2017 were at the stage 3B of gonad development, during which the gonads of diploid oysters were dramatically developed morphologically ripe, covering all of the visceral mass, the soft parts were extremely full, and the gametes immediately scattered when put into seawater. The gonads of oysters collected in December 2016 were at stage 0. At this stage, the gonads of the oysters remained inactive and undifferentiated, and the soft parts were clear and colorless (Allen and Downing, 1986; Ojea et al., 2004; Dridi et al., 2007).

According to the protocols developed by Downing and Allen (1987) and Qin et al. (2018), the ploidy status of each individual oyster was determinated using flow cytometry using a CyFlow Ploidy Analyser (Sysmex). Then, subsamples of gill, mantle, adductor muscle, labial palps, and gonad tissue were stored at −80°C for glycogen and protein content analysis, and all tissues were lyophilized before use. The tissues were mingled to create mixed samples from 10 individuals of the same ploidy status. The same individuals used for glycogen content determination were also used for related gene expression analysis. Five tissue samples were immediately dissected from each oyster, placedin 1 mL Trizol and wholly ground with a homogenizer, then frozen at −80°C. For amino acid and fatty acid analysis of the whole oysters, three pools each of 10 diploids and 10 triploids were collected and immediately dissected, then freeze dried. All analyses were done in triplicate.

### Glycogen and protein content analyses

The glycogen content of the five lyophilized tissues of diploids and triploids, was analyzed using a kit for detecting glycogen content (Nanjing Jiancheng Bioengineering Institute).The kit was used according to the following procedure: after 1 mg dry weight (DW) of tissue was ground into a powder in the presence of liquid nitrogen, a 0.50 μg sample was added to a tube containing alkaline liquid. Then, the tube was heated at 100°C for 20 min in a water bath. After that, the hydrolysate was diluted 16-fold by adding sterile water. Subsequently, 2 mL of color reagent was added to the diluted hydrolysate and the samples were heated for 5 min at a 100°C in a water bath. Finally, the OD value of each sample was measured at 620 nm using a Multimode Plate Reader (Ensight) after setting the blank and standard for each group. The glycogen content was calculated according to the following formula:

$$\begin{array}{l} \text{Glycogen content (mg/g, DW)}\\ \;=(\frac{\text{OD of test group}-\text{OD of blank group}}{\text{OD of standard group}-\text{OD of blank group}})\\ \;\times 0.01\times 20\times 10÷1.11 \end{array}$$

For protein content analyses, the lyophilized tissues were wholly ground with a homogenizer and then analyzed using a fully automatic Kjeldahl analyzer (PeiOU, Skd-800). Finally, protein content was calculated by multiplying nitrogen content by a factor of 6.25 (Iqbal et al., 2006).

### Amino acid and fatty acid content assays

Amino acid analyses were performed according to the method of Chen and Zhang (Hugli and Moore, 1972; Chen and Zhang, 2007; Chen et al., 2007). Lyophilized meat samples of diploid and triploid Hong Kong oysters (three pools of 10 oysters from each group) were weighed (0.0150–0.0250 g), placed in 15 mL ampoules, and 8 mL of (6.0 N) HCl was added. Ampoules were vacuum sealed, and samples were hydrolyzed at 110°C for 24 h. Following hydrolysis, 1 mL of hydrolyzate was withdrawn and evaporated to dryness under vacuum at 45°C to remove HCl. The hydrolyzate was dissolved in 5 mL of 0.02 N HCl, and then centrifuged at 5,000 rpm and filtered. One microliter of supernatant was used for amino acid analysis. The identity and quantity of the amino acids were determined by comparison with the retention times and peak areas of each amino acid standard. The tryptophan content was determined by Guangdong Food Quality Supervision Inspection Station according to GB 5009.168-2016.

Fatty acids were analyzed according to Doan et al. (2018). In brief, 10 mg of dry tissue was dissolved in 1 mL of petroleum ether. Then, 25 μL of 2 M sodium methanolate methanol solution was added, and the closed vial was agitated vigorously for 1 min. About 20 μL of water was added, and after centrifugation the aqueous phase was removed. Then 20 μL methyl orange in 0.1 N HCl was added as a pH indicator. The mixture was agitated carefully and the derivatives were analyzed using a Hewlett-Packard Gas Chromatography Instrument.

### Real-time quantitative PCR analysis of genes

According to the sequence of the *C. gigas* glycogen synthesis gene (Genbank accession bank number: NM001308922.1), glycogen phosphorylase gene (Genbank accession bank number: NM001305341.1), citrate synthase gene (Genbank accession bank number: XM011429904.2), and α-ketoglutarate dehydrogenase gene (Genbank accession bank number: XM011437307.2), and a BLAST analysis of all EST sequences from a *C. hongkongensis* hemocyte EST library which was constructed and sequenced by our lab, the open reading frames (ORF) of *Ch*GS, *Ch*GP, *Ch*CS, and *Ch*KD have already been obtained. The temporal expression of the genes in various tissues (gill, mantle, labial palps, muscle, and gonad) of the oysters during the reproductive and non-reproductive phases were investigated using real-time quantitative PCR (qPCR) analysis. We designed gene-specific primers (Table 1) based on ORF to amplify gene fragments ~250 bp in length. Amplifications of actin and elongation factor 1α gene (EF1α) fragments were performed in order to confirm the steady-state level of expression of housekeeping genes, providing an internal reference for gene expression (Zhou et al., 2018).

**Table 1:** Sequences of designed primers used in this study.

**Primer**	**Sequence (5′ → 3′)**	**Comment**
*Ch*GS-F	TCGGCTGGGAGATATGAGTT	qPCR of *Ch*GS
*Ch*GS-R	AGTTGTTGGTCTTGGTGGGG
*Ch*GP-F	ACCACGAGAAGCGAAAGCAAATCAG	qPCR of *Ch*GP
*Ch*GP-R	CGGTGTCGCCACATTTCTATCTTTC
*Ch*KD-F	TACTCTGGCTCGTCTCGT	qPCR of *Ch*KD
*Ch*KD-R	GCACATTCAGTCTCCCTCT
*Ch*CS-F	TCTCCTCTCCCCAACCAT	qPCR of *Ch*CS
*Ch*CS-R	TAATGGCAGCAATGGTGG
EFLα-F	CGGGATCCATGTATAGTCGGGAGA	qPCR of EFLα
EFLα-R	CCCAAGCTTTCACAGAGAAATCAA

“*F” indicates forward primers and “R” indicates reverse primers*.

The real-time PCR amplifications were carried out in triplicate in a total volume of 10 μL with 5 μL of 2 × Real Star Green Power mixture (Genstar), 0.50 μM each of forward and reverse primers, 1 μL of the 1:20 diluted cDNA using Light Cycler 480II (Roche). The PCR protocol was: 95°C for 10 min, followed by 40 cycles of 95°C for 15 s, 60°C for 30 s, and 72°C for 30 s. The relative expression ratio (R) of each target gene was calculated based on the threshold value (Ct) deviation of this target gene from the housekeeping gene, corresponding to the copy number of the target gene relative to the copy number of the housekeeping gene. So, the following formula was used:

$$\begin{array}{l} \text{R}=2^{-(Ct_{target\;gene}-Ct_{housekeeping\;gene})} \end{array}$$

### Statistical analysis

All data are presented as mean ± standard deviation (SD). Multiple comparisons of the relative expression levels of target genes and biochemical makeup of different tissues from diploid and triploid oysters during the reproductive and non-reproductive phases were performed using one-way analysis of variance (ANOVA) followed by a multiple comparison test with the LSD-*t*-test using Statistical Package for the Social Sciences (SPSS18). *P* < 0.05 was considered significant, while *P* < 0.01 was considered extremely significant.

The odds ratio (OR) is defined as the percent difference in the biochemical components (glycogen, fatty acid, and amino acid contents) between the diploid (2N) and the triploid oyster (3N) performances and is calculated using the following formula (Callam et al., 2016):

$$\begin{array}{l} \text{OR}\left(\%\right)=\left(\frac{3N-2N}{2N}\right)\times 100 \end{array}$$

where a positive OR indicates that the triploid oysters performed better than the diploids, and a negative OR indicates that the triploids performed worse.

The essential amino acid score was calculated with reference to the FAO/WHO reference amino acid pattern for preschool children (2–5 years old) (FAO/WHO/UNU, 1985), according to the following formula:

$$\begin{array}{l} \text{Amino acid score}=\left(\frac{\text{Test amino acid}}{\text{Reference amino acid}}\right)\times 100 \end{array}$$

The reason for using the requirements of young children as a reference is that, if the amino acid composition of proteins can adequately support the healthy growth of preschool children, it will be more than adequate for adults (Kirimura et al., 1969; Gatenby et al., 2003).

## Results

### Glycogen content and *Ch*GS and *Ch*GP mRNA expressions

**Figure 1:**
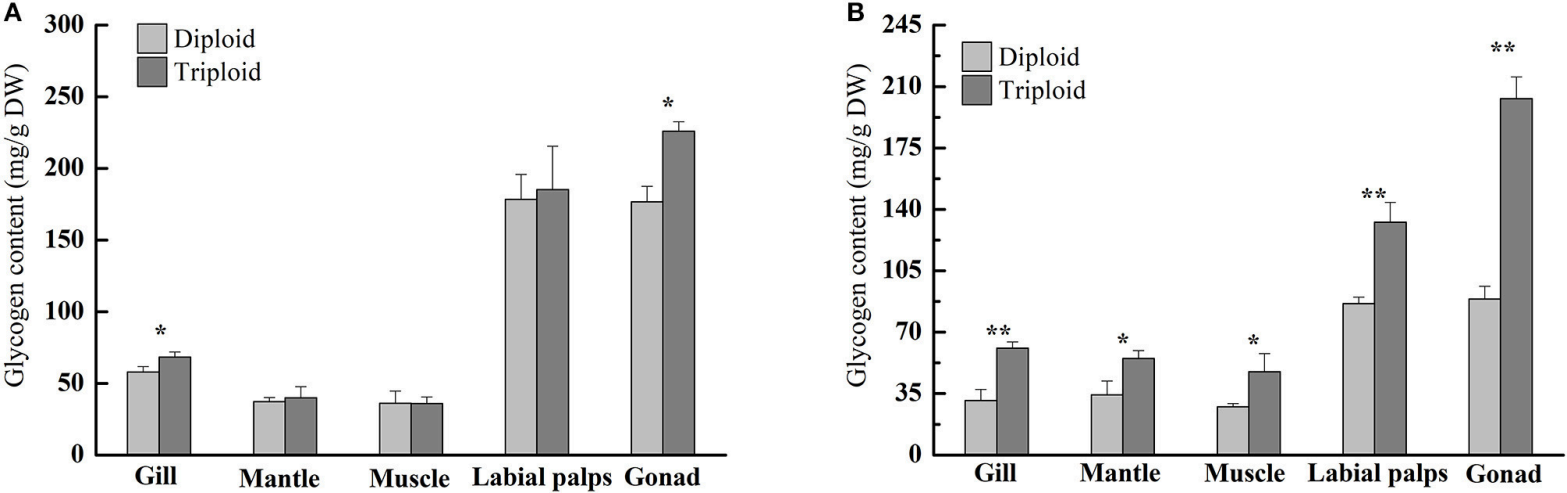
Glycogen contents (mg/g, DW) of various tissues (gill, mantle, muscle, labial palps, and gonad) of diploid and triploid Hong Kong oysters, *Crassostrea hongkongensis*, in the non-reproductive phase **(A)** and reproductive phase **(B)**. Significant differences are indicated by asterisks (^*^ and ^**^ represent *P* < 0.05 and *P* < 0.01, respectively).

In the non-reproductive phase (December), the average glycogen content of diploids was 36.20 ± 8.59 mg/g DW in the muscle, 37.25 ± 2.98 mg/g DW in the mantle, 57.89 ± 3.88 mg/g DW in the gill, 176.69 ± 10.80 mg/g DW in the gonad, and 178.49 ± 17.41 mg/g DW in the labial palps. In triploids, the average glycogen content was 35.93 ± 4.55 mg/g DW in the muscle, 39.96 ± 7.78 mg/g DW in the mantle, 68.35 ± 3.62 mg/g DW in the gill, 185.19 ± 30.42 mg/g DW in the labial palps, and 226.03 ± 6.70 mg/g DW in the gonad (Figure 1A). There were statistically significant differences in gill and gonad glycogen content between diploids and triploids (*P* < 0.05), with ORs of 18.07 and 27.92%, respectively (Table 2). In the non-reproductive phase, triploids showed highly significant elevations in ChGS and ChGP expressions in tissues compared to diploids (*P* < 0.01) (**Figures 3A,B**).

**Table 2:** The odds ratios (ORs) of the glycogen and protein content of various tissues (gill, mantle, muscle, labial palps, and gonad) between diploid and triploid Hong Kong oysters, *Crassostrea hongkongensis*, in the non-reproductive and reproductive phases.

			**Gill**	**Mantle**	**Muscle**	**Labial palps**	**Gonad**
OR (%)	Glycogen	Non-reproductive	18.07	7.28	−0.75	3.75	27.92
		Reproductive	96.26	60.17	72.59	53.56	128.52
	Protein	Non-reproductive	−0.72	3.14	−1.67	1.60	0.52
		Reproductive	1.99	−0.22	−2.00	0.97	−11.41

However, in the reproductive season (July), the average glycogen content of diploids was 27.47 ± 1.81 mg/g DW in the muscle, 31.00 ± 6.45 mg/g DW in the gill, 34.30 ± 7.84 mg/g DW in the mantle, 86.39 ± 3.63 mg/g DW in the labial palps, and 88.87 ± 7.24 mg/g DW in the gonad. In triploids, the average glycogen content was 47.41 ± 10.46 mg/g DW in the muscle, 54.94 ± 4.55 mg/g DW in the mantle, 60.84 ± 3.64 mg/g DW in the gill, 132.66 ± 11.26 mg/g DW in the labial palps, and 203.09 ± 12.41 mg/g DW in the gonad. The glycogen content of all five tissues was significantly higher (*P* < 0.05) in triploids than in diploids (Figure 1B). The ORs of the five tissues (gill, mantle, muscle, labial lalps, and gonad) were 96.26, 60.17, 72.59, 53.56, and 128.52%, respectively (Table 2). In addition, we found that the glycogen content of some tissues was lower during the reproductive season than in the non-reproductive phase in diploid oysters, especially in the labial palps and gonad, which had ORs of −51.60 and −49.70% comparing the reproductive to the non-reproductive phase. By contrast, from the non-reproductive to the reproductive phase, the five triploid tissues had glycogen content ORs of −10.99, 37.49, 31.95, −28.37, and −10.15% (Table 3). In the reproductive phase, significantly higher (*P* < 0.05) expressions of *Ch*GS were observed in triploids than in diploids. In contrast, the expressions of *Ch*GP were significantly lower (*P* < 0.05) in triploids (**Figures 4A,B**).

**Table 3:** The odds ratios (ORs) of the glycogen and protein content of various tissues (gill, mantle, muscle, labial palps, and gonad) of diploid and triploid Hong Kong oysters, *Crassostrea hongkongensis*, between the non-reproductive phase and the reproductive phase.

			**Gill**	**Mantle**	**Muscle**	**Labial palps**	**Gonad**
OR (%)	Glycogen	2N	−46.45	−7.92	−24.12	−51.60	−49.70
		3N	−10.99	37.49	31.95	−28.37	−10.15
	Protein	2N	1.03	8.57	0.18	3.49	17.72
		3N	3.79	5.03	−0.16	2.84	3.75

### Protein content

**Figure 2:**
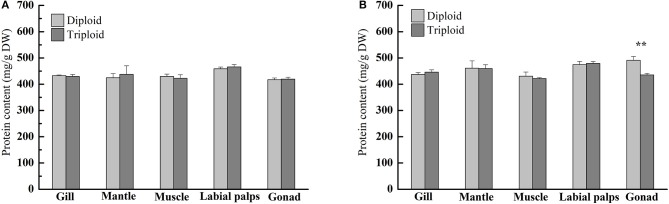
Protein contents (mg/g, DW) of various tissues (gill, mantle, muscle, labial palps, and gonad) of diploid and triploid Hong Kong oysters, *Crassostrea hongkongensis*, in the non-reproductive phase **(A)** and reproductive phase **(B)**. Significant differences are indicated by asterisks (^**^ represent *P* < 0.01).

According to our research, in the non-reproductive stage, the protein content ranged from 424.46 ± 16.00 (mantle) to 458.73 ± 6.77 mg/g DW (labial palps) in diploids and from 422.47 ± 13.78 (muscle) to 466.07 ± 8.87 mg/g DW (labial palps) in triploids, and there were no statistically significant differences in protein content between diploid and triploid oysters (Figure 2A, Table 2). In the reproductive phase, the protein content was lowest in the muscle tissue of both diploids and triploids, with 430.84 ± 16.05 and 421.79 ± 3.52 mg/g DW, respectively. Conversely, the protein content of diploid and triploid oysters was highest in the gonad and labial palps, respectively, with 491.23 ± 14.38 and 479.32 ± 7.23 mg/g DW. In addition, except in the gonad (*P* < 0.05), there were no significant differences in the protein content between diploids and triploids in the reproductive phase (Figure 2B, Table 2).

### Amino acid and fatty acid content

During the non-reproductive phase, the individual amino acid levels were highest for glutamic acid and lowest for methionine in both diploid and triploid oysters (Table 4). In the reproductive phase, the diploid Hong Kong oysters contained glutamic acid in the largest amounts (142.0 mg/g of protein), followed by aspartic acid, lysine, leucine, arginine, and glycine in descending order. Individual amino acid contents ranged from 20.2 (methionine) to 153.8 mg/g of protein (glutamic acid) in triploids (Table 4). When compared to the reference amino acid pattern of children, all of the essential amino acid scores in diploids and triploids were more than 100, except that of methionine, no matter in the non-reproductive phase or the reproductive phase (Table 5).

**Table 4:** Amino acid composition of diploid and triploid Hong Kong oysters, *Crassostrea hongkongensis*, in the non-reproductive and reproductive phases.

	**Non-reproductive**				**Reproductive**			
**Amino acid**	**Content (g/100 g meat)**		**Content (mg/g protein)**		**Content (g/100 g meat)**		**Content (mg/g protein)**	
	**2N**	**3N**	**2N**	**3N**	**2N**	**3N**	**2N**	**3N**
Aspartic acid	4.16 ± 0.60	4.27 ± 0.57	107.8	108.4	5.83 ± 0.98*a*	3.91 ± 0.07*b*	111.3	105.1
^*^Threonine	1.92 ± 0.27	1.93 ± 0.24	49.7	49.0	2.72 ± 0.48*a*	1.77 ± 0.04*b*	51.9	47.6
Serine	2.04 ± 0.25	2.00 ± 0.36	52.8	50.8	2.95 ± 0.64*a*	1.80 ± 0.03*b*	56.3	48.4
Glutamic acid	5.43 ± 0.66	5.86 ± 0.25	140.7	148.7	7.44 ± 1.30	5.72 ± 0.19	142.0	153.8
Proline	1.67 ± 0.28	1.81 ± 0.08	43.3	45.9	2.22 ± 0.29	1.76 ± 0.04	42.4	47.3
Glycine	2.46 ± 0.52	2.94 ± 0.38	63.7	74.6	2.97 ± 0.52	3.14 ± 0.04	56.7	84.4
Alanine	2.09 ± 0.26	2.24 ± 0.08	54.1	56.9	2.75 ± 0.48*a*	2.20 ± 0.04*b*	52.5	59.1
^*^Valine	2.07 ± 0.30	2.03 ± 0.30	53.6	51.5	2.88 ± 0.48*a*	1.83 ± 0.04*b*	55.0	49.2
^*^Methionine	0.71 ± 0.13	0.77 ± 0.03	18.4	19.5	0.95 ± 0.11*a*	0.75 ± 0.03*b*	18.1	20.2
^*^Isoleucine	1.73 ± 0.27	1.73 ± 0.27	44.8	43.9	2.48 ± 0.45*a*	1.55 ± 0.03*b*	47.3	41.7
^*^Leucine	2.78 ± 0.35	2.67 ± 0.41	72.0	67.8	3.92 ± 0.75*a*	2.45 ± 0.04*b*	74.8	65.9
Tyrosine	1.36 ± 0.13	1.38 ± 0.14	35.2	35.0	1.83 ± 0.36	1.31 ± 0.07	34.9	35.2
^*^Phenylalanine	2.01 ± 0.21	1.94 ± 0.32	52.1	49.2	2.87 ± 0.63*a*	1.74 ± 0.03*b*	54.8	46.8
^*^Lysine	3.18 ± 0.35	2.91 ± 0.51	82.4	73.9	4.32 ± 0.79*a*	2.63 ± 0.02*b*	82.4	70.7
^*^Histidine	0.94 ± 0.20	0.99 ± 0.16	24.4	25.1	1.22 ± 0.09*a*	0.92 ± 0.08*b*	23.3	24.7
^*^Arginine	2.74 ± 0.27	2.62 ± 0.28	71.0	66.5	3.71 ± 0.72*a*	2.46 ± 0.02*b*	70.8	66.1
Tryptophan	1.31 ± 0.02	1.32 ± 0.04	33.9	33.5	1.34 ± 0.10	1.26 ± 0.05	25.6	33.9
Total	38.60	39.41	999.9	1000.2	52.40	37.16	1000.1	999.0

*Means ± SD followed by different letter within a line are significantly different (P < 0.05)*.

“*” *indicate essential amino-acids*.

**Table 5:** Essential amino acid scores of diploid and triploid Hong Kong oysters, *Crassostrea hongkongensis*, in the non-reproductive and reproductive phases.

		**Non-reproductive**		**Reproductive**	
**Amino acid**	**Reference(mg/g protein)**	**Score 2N**	**Score 3N**	**Score 2N**	**Score 3N**
Threonine	34	146	144	153	140
Valine	35	153	147	157	141
Methionine	25	74	78	72	81
Isoleucine	28	160	157	169	149
Leucine	66	109	103	113	100
Phenylalanine + tyrosine	63	138	134	142	130
Lysine	58	142	127	142	122
Histidine	19	128	132	123	130
Tryptophan	11	308	304	233	308
Total	339			

*Reference amino acid pattern for preschool children (2–5 years) (FAO/WHO/UNU, 1985). Each amino acid in the reference was presumed to score a value = 100. Score for each amino acid is expressed relative to the reference*.

In the non-reproductive phase, 21 fatty acids were present in diploids and triploids. The proportion of saturated fatty acids (SFAs) was lower in triploids than in diploids, while that of PUFAs was higher, indicating that triploids have a better fatty acid composition for human consumption than diploids during the non-reproductive phase. In diploids, the proportions of individual PUFAs making up the total PUFA content ranged from 0.16 ± 0.01% for γ-linolenic acid (18:3n-6) to 11.28 ± 1.05% for DHA (22:6n-3). In triploids, the dominant PUFA was also DHA (22:6n-3), accounting for 14.30 ± 0.39% of the total fatty acid content, and there appeared to be more n-3 than n-6 fatty acids (Table 6). During the reproductive phase, 19 fatty acids were found in diploid and triploid Hong Kong oysters. The fatty acid compositions of the diploids and triploids in the reproductive phase are shown in Table 6. The fatty acid profiles of both diploids and triploids were dominated by SFAs, which made up 58.26 and 55.34% of the total fatty acids, respectively. Among the SFAs, the proportions of fatty acids in diploids ranged from 0.10 ± 0.03% for lauric acid (12:0) to 38.35 ± 0.74 for palmitic acid (16:0), which was also the dominant SFA in triploids (37.6 ± 2.00% of the total fatty acids). EPA (20:5n-3) was the dominant PUFA in both diploids and triploids, followed by DHA (22:6n-3), ALA (18:3n-3), and LA (18:2n-6) in descending order. Triploids had a greater PUFA content than diploids, with PUFAs making up 37.71% of the total fatty acid content of triploids.

**Table 6:** Fatty acid composition (% of total fatty acids) of diploid and triploid Hong Kong oysters, *Crassostrea hongkongensis*, in the non-reproductive and reproductive phases.

**Fatty acid**	**Non-reproductive**		**Reproductive**	
	**Mean ± SD/2N**	**Mean ± SD/3N**	**Mean ± SD/2N**	**Mean ± SD/3N**
**SATURATED FATTY ACID (SFA) COMPOSITION (%)**				
8:0	–	–	0.25 ± 0.01	0.29 ± 0.05
12:0	–	–	0.10 ± 0.03	0.09 ± 0.02
14:0	3.04 ± 0.10	2.97 ± 0.09	6.13 ± 0.10a	3.42 ± 0.28b
15:0	1.42 ± 0.04	1.32 ± 0.04	0.98 ± 0.07	1.24 ± 0.17
16:0	38.13 ± 0.60a	33.7 ± 0.04b	38.35 ± 0.74	37.6 ± 2.00
17:0	4.25 ± 0.26a	3.79 ± 0.04b	2.64 ± 0.27	2.99 ± 0.21
18:0	11.23 ± 0.31a	9.84 ± 0.33b	9.53 ± 0.37	9.42 ± 0.67
20:0	0.21 ± 0.02	0.19 ± 0.01	0.28 ± 0.01	0.29 ± 0.03
21:0	0.10 ± 0.01	0.10 ± 0.01	–	–
22:0	0.34 ± 0.01	0.33 ± 0.01	–	–
ΣSFA	58.72	52.24	58.26	55.34
**MONOUNSATURATED FATTY ACID (MUFA) COMPOSITION (%)**				
16:1n-7	1.95 ± 0.06	1.94 ± 0.11	4.46 ± 0.50	3.97 ± 0.47
18:1n-9	5.39 ± 0.05	5.41 ± 0.02	4.42 ± 0.47	5.09 ± 0.30
20:1n-9	0.90 ± 0.03	0.83 ± 0.05	–	–
22:1n-9	1.28 ± 0.10a	0.98 ± 0.07b	–	–
ΣMUFA	9.52	9.16	8.88	9.06
**POLYUNSATURATED FATTY ACID (PUFA) COMPOSITION (%)**				
18:2n-6	2.49 ± 0.12a	2.79 ± 0.05b	2.57 ± 0.11a	3.25 ± 0.06b
18:3n-6	0.16 ± 0.01a	0.18 ± 0.01b	0.33 ± 0.02a	0.46 ± 0.02b
18:3n-3	2.88 ± 0.39a	3.85 ± 0.09b	4.25 ± 0.31a	5.13 ± 0.35b
20:2n-6	0.40 ± 0.01	0.41 ± 0.02	0.40 ± 0.04	0.35 ± 0.02
20:3n-6	0..22 ± 0.01	0.21 ± 0.01	0.28 ± 0.03	0.28 ± 0.01
20:4n-6	3.43 ± 0.08	3.35 ± 0.12	2.13 ± 0.52	2.01 ± 0.03
20:3n-3	0.20 ± 0.01	0.22 ± 0.02	0.24 ± 0.03	0.23 ± 0.02
20:5n-3	10.49 ± 0.71a	14.27 ± 0.83b	13.4 ± 0.44a	15.07 ± 0.71b
22:6n-3	11.28 ± 1.05a	14.30 ± 0.39b	8.61 ± 0.24a	10.93 ± 0.84b
ΣPUFA	31.55	39.58	32.21	37.71
ΣPUFA n-3	24.85	32.64	26.50	31.36
ΣPUFA n-6	6.70	6.94	5.71	6.35
ΣPUFA n-3/ΣPUFA n-6	3.71	4.70	4.64	4.94
ΣPUFA n-6/ΣPUFA n-3	0.27	0.21	0.22	0.20

*Means ± SD followed by different letter within a line are significantly different (P < 0.05). “–” indicates < 0.0033%*.

## Discussion

### The glycogen content variation pattern is correlated with ChGS and ChGP mRNA expression patterns

According to our study, in the non-reproductive phase, there were statistically significant differences in gill and gonad glycogen content between diploids and triploids, as the glycogen content of triploid Hong Kong oysters increased in response to increasing translatable mRNA of *Ch*GS, especially in the labial palps and gonad. The same phenomenon has been observed in *C. gigas* and *C. virginica* (Bacca et al., 2005; Zeng et al., 2013; Guévélou et al., 2017). Pogoda et al. (2013) confirmed that *O. edulis* and *C. gigas* juveniles and adults primarily utilized glycogen to store energy, and that all tissues were capable of glycogen hydrolysis and glucose formation to provide ATP for growth. This process is known to be modulated by variations in *Ch*GS and *Ch*GP expressions. In our study, the rates of *Ch*GS and *Ch*GP transcription appeared to vary depending on the tissue and ploidy status. In the non-reproductive phase, highly significant differences (*P* < 0.01) in levels of *Ch*GS expression existed between diploid and triploid oysters in all five tissues. In the mantle, muscle, labial palps, and gonad, there were also very significant differences (*P* < 0.01) in *Ch*GP expression between diploid and triploid oysters. According to Dégremont et al. (2012) and Zhang et al. (2017), triploid oysters had a faster growth rate than diploids, and therefore had higher energy requirements for growth. Because of the faster growth in triploid Hong Kong oysters, the tissue-specific expressions of *Ch*GS and *Ch*GP (except in the gill) have been shown to be very significantly higher (*P* < 0.01) in triploids than in diploids (Zhang et al., 2017).

In the present study, significantly higher (*P* < 0.05) expressions of *Ch*GS in triploids were observed across the five tissues in the reproductive season, but expressions of *Ch*GP in all tissues were significantly lower (*P* < 0.05) in triploids than in diploids. This is in accordance with the higher glycogen content observed in all five tissues of triploid oysters. *Ch*GS and *Ch*GP mRNA expressions are closely related to glycogen content, as previously characterized in *C. gigas* (Berthelin et al., 2000a,b), so it is believed that the observed variations in *Ch*GS and *Ch*GP mRNA levels in diploid *C. hongkongensis* are closely linked to the stages of reproduction. Additionally, the OR of the glycogen content of the gonad of triploid oysters from the non-reproductive to the reproductive phase was much lower than that of diploids, indicating that much less glycogen was required to support gonad development in triploid Hong Kong oysters in the reproductive phase. Zwaan and Zandee (1972) and Berthelin et al. (2000a,b) reported the same phenomenon, finding that the storage and mobilization of glycogen was correlated with the reproductive cycle of bivalves. The glycogen storage levels of *Mytilus edulis* and *C. gigas* were lowest during the reproductive phase, while phytoplanktonic food was potentially abundant, suggesting that diploids were unable to store reserves at this time due to gametogenesis. Based on Zhang et al. (2017) and Qin et al. (2018), the gonad development and spawning stage of diploid Hong Kong oysters lasts from May to August. In our study, the diploids went through the maturation stage until July, and needed to consume large amounts of glycogen to support gonad development. In male diploid oysters, energy in the forms of glycogen would be mobilized for active production of germ cells, while in diploid females glycogen would be mobilized for vitellogenesis due to oocyte maturation, with the accumulation of yolk and other nutritive substances (Fabioux et al., 2004; Bacca et al., 2005; Zeng et al., 2013). In addition, according to Allen and Downing (1986) and Jeung et al. (2016), reproductive sterility is a common feature of triploid oysters as a result of the extra set of chromosomes, and gonadogenesis in triploids is either reduced or absent, with reduced numbers of gametes even if a small number of triploids are fertile.

From our results, together with previous studies, we conclude that in both diploid or triploid Hong Kong oysters, expressions of *Ch*GS and *Ch*GP appear to be related to glycogen content, indicating that their expressions are likely to be involved in glycogen regulation, as observed in other bivalves and mammals (Zwaan and Zandee, 1972; Towle, 1995; Berthelin et al., 2000a; Vali et al., 2000). Glycogen content is an important factor that affects taste, meaning that sterile triploid Hong Kong oysters have added value, because their increased glycogen content allows them to be marketed during the reproductive phase, when diploids suffer from low meat quality.

### Crude protein and amino acid contents

Proteins are the fundamental building blocks for tissue biosynthesis and enzyme production in all animals. Dietary protein must meet the demands for tissue production and metabolic processes, and meat consumption is an important part of this for most consumers (Manninen et al., 2018). High-quality protein is generally considered most important for rapidly growing young chindren, followed by adults undergoing gametogenesis (Kirimura et al., 1969; Gatenby et al., 2003). High-quality protein content directly contributes to the nutritional value of food, and has a large effect on the market value. In the reproductive phase, there were no statistically significant differences in protein content between diploid and triploid oysters, which may indicate that the overwhelming majority of ATP supporting the fast growth of triploid Hong Kong oysters was produced thorugh glycogen metabolism. However, there was a significant difference (*P* < 0.05) between diploids and triploids in the protein content of the gonad, in agreement with a previous study done in *C. gigas* by Ren et al. (2003), in which gonad protein levels correlated positively with the gametogenic cycle, increasing with gonad development and oocyte diameter. Together with Dare and Edwards (1975), they concluded that glycogen and lipid stored in the mantle and gonad contributed to the increase in protein content in the gonad during the reproductive phase. Because of their sterility, the OR of triploids (3.75%) was smaller than that of diploids (17.72%) from the non-reproductive to the reproductive phase (Table 3). Based on the experiments, protein was typically the major biochemical component of diploid and triploid Hong Kong oysters and maintained a relatively stable levels (consistently above 40% DW) compared to glycogen between the reproductive and non-reproductive phases, similar to diploid *C. gigas* (Ren et al., 2003), *O. edulis* (Ruiz et al., 1992), and *M. edulis* (Dare and Edwards, 1975). In general, we confirmed that both diploid and triploid oysters were high protein foods regardless of the phase of the reproductive cycle.

Amino acids are associated with flavors of sweetness, bitterness, sourness, saltiness, and umami, and are very important in contributing to the taste of foodstuffs. Protein quality is determined based on levels of essential amino acids for humans (Kirimura et al., 1969). In the non-reproductive phase, there were no significant differences between the levels of individual amino acids in diploid and triploid oysters. In both diploids and triploids, the methionine content was the lowest of the essential amino acids, and had scores of <100 when compared to the reference amino acid pattern for preschool children (2–5 years old) (FAO/WHO/UNU, 1985), indicating that methionine was the limiting amino acid in both diploid and triploid Hong Kong oysters (Table 5). Glutamic acid and aspartic acid were found to be major non-essential amino acids in diploids and triploids, as reported in *C. gigas* (Kong et al., 2001) and *M. edulis* (Su et al., 1998). In the reproductive phase, significant differences (*P* < 0.05) existed in the individual essential amino acid levels between diploids and triploids, particularly for valine, isoleucine, leucine, phenylalanine, and lysine, which were higher in diploids and had ORs of −8.01, −6.78, −10.53, −12.38, and −11.50%, respectively. This is likely related to gonad development, as reported in *C. gigas* by Kong et al. (2001). Kong et al. (2001) also found that the amino acid composition of diploid *C. gigas* changed dramatically with gonad development, but remained relatively stable in triploids. According to the reference amino acid pattern, methionine levels did not meet the recommendations for preschool children in either diploids or triploids (scored < 100); however, given that about 55% of methionine is lost when meat was hydrolyzed without performic acid oxidation, the true methionine score would likely be over 100 (Spindler et al., 1985; Chen et al., 2007). Overall, diploid and triploid Hong Kong oysters are high protein foods and are relatively well-balanced in terms of essential amino acid composition, which indicates that they are a high-quality protein source.

### Fatty acid content

Essential fatty acids, namely ALA, LA, EPA, and DHA, are very important for human health (Coetzee and Hoffman, 2002). ALA and DHA are major components of cell membrane phospholipids and are the predominant long-chain PUFAs of the central nervous system (Dyerberg, 1986). The ratio between n-3 and n-6 long chain PUFAs (n-3/n-6) is also considered to be important (Coetzee and Hoffman, 2002; Simopoulos, 2008). Lack of n-3 PUFAs and very low n-3/n-6 ratios promote the pathogenesis of many diseases, including cancer, inflammatory, and autoimmune diseases (Park et al., 1994). In both the non-reproductive and the reproductive phases, the proportion of saturated fatty acids (SFAs) was lower and that of polyunsaturated fatty acids (PUFAs) was higher in triploids than in diploids, indicating that triploids have better fatty acid composition as a human food source (Chen and Zhang, 2007). In both phases, significant differences (*P* < 0.05) were observed between diploids and triploids in the proportions of LA, ALA, γ-linolenic acid, EPA, and DHA. EPA and DHA were the dominant PUFAs. Many researchers have also confirmed that variations in the fatty acid composition of bivalves were related to gametogenic cycle, such as in *C.gigas* (Dridi et al., 2007), *Subnodosus nodipecten* (Racotta et al., 2008), *R. decussatus* (Ojea et al., 2004), and *M. galloprovincialis* (Orban et al., 2002).

Furthermore, the proportion of n-3 PUFAs was significantly higher than that of n-6 in both diploid and triploid Hong Kong oysters. An increase in the levels of n-3 PUFAs in the triploid oysters effectively reduced the proportion of SFAs, making them healthier for human consumption (Celik et al., 2004). There is no question that n-3 PUFAs are important in the human diet, or that they are found in a wide variety of foods. The n-3/n-6 ratio is a good index for comparing the relative nutritional value of foods from different sources, and a high n-3/n-6 PUFA ratio has often been cited as an indicator of high nutritional value (Skonberg and Perkins, 2002). The n-3/n-6 PUFA ratio of triploid Hong Kong oysters was higher than that of diploids in both the non-reproductive (4.70 and 3.71, respectively) and reproductive (4.94 and 4.64, respectively) phases. By contrast, in *C. gigas*, the n-3/n-6 PUFA ratio was only 3.15 in the non-reproductive stage and only 2.45 in the reproductive stage (Dridi et al., 2007). The difference in fatty acid composition and the n-3/n-6 PUFA ratio between *C. gigas* and *C. hongkongensis* can be attributed to special biological traits (endogenous origins) and living environments (exogenous origins). Orban et al. (2002) confirmed a close relationship between temperature and PUFA compositions in *M. galloprovincialis*, and Napolitano et al. (1997) reported that seasonal variations in fatty acid composition of *Placopecten magellanicus* reflected the fatty acid composition of the phytoplankton. Precisely the opposite from *C. gigas, C. hongkongensis* live in a high temperature and low salt sea area. As *C. hongkongensis* grow slowly, it usually takes 3 years to reach the specified commercial size (Qin et al., 2018). Dyerberg noted that an increase in the ratio of n-3/n-6 PUFAs increases the availability of n-3 PUFAs, which are beneficial for human health (Dyerberg, 1986). In general, triploid Hong Kong oysters have higher nutritional value than diploids, since the ratio of n-3/n-6 PUFAs is higher than diploids.

### The tricarboxylic acid cycle related to variations in biochemical composition

**Figure 3:**
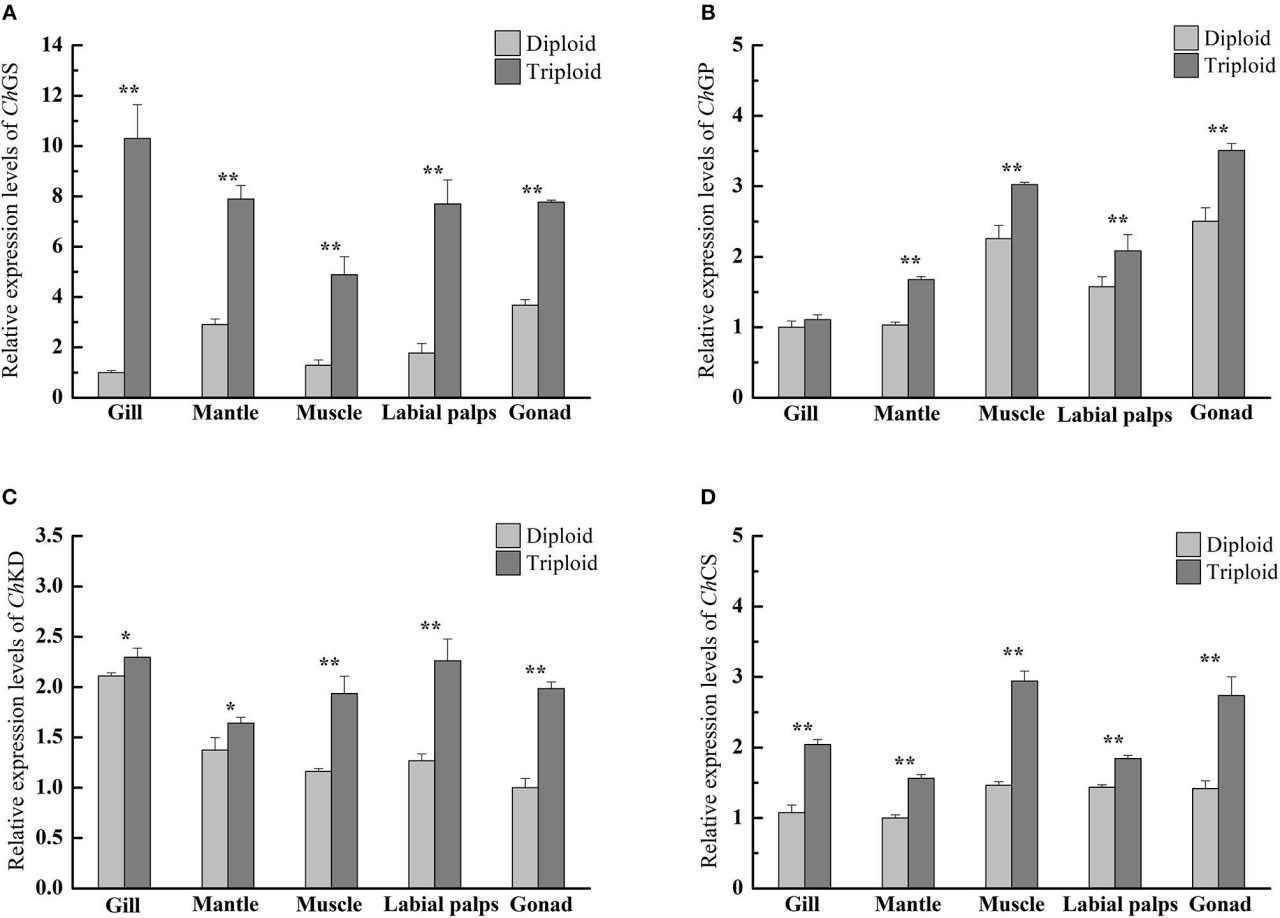
Quantitative PCR analysis of relative mRNA expressions of *Ch*GS **(A)**, *Ch*GP **(B)**, *Ch*KD **(C)**, and *Ch*CS **(D)** in various tissues (gill, mantle, muscle, labial palps, and gonad) of diploid and triploid Hong Kong oysters in the non-reproductive phase. Each bar represents the mean of the normalized expression levels of the replicates (*n* = 3). Significant differences are indicated by asterisks (^*^ and ^**^ represent *P* < 0.05 and *P* < 0.01, respectively).

The tricarboxylic acid (TCA) cycle is the final metabolic pathway of glycogen, lipids and proteins, and is also a hub for the metabolism of three major nutrients. The biochemical composition of organisms is closely related to the TCA cycle. *Ch*CS and *Ch*KD are the key enzyme genes in the TCA cycle. According to our research, during the non-reproductive phase, the relative expressions of *Ch*KD and *Ch*CS were significant higher (*P* < 0.05) in all five tissues (gill, mantle, muscle, labial palps, and gonad) of triploids than in those of diploids (Figures 3C,D). Interestingly, the glycogen content was significantly higher in the gill and gonad of triploids than those of diploids, while there was no signiciant difference in protein content between triploids and diploids. Although the high levels of expression of *Ch*GS and *Ch*GP were related to glycogen metabolism, higher expressions of *Ch*KD and *Ch*CS in triploid oysters revealed that these oysters have a higher matabolic rate than diploid oysters, which may contribute to their faster growth. The same phenomenon was discovered by Li et al. (2017), who found that the TCA cycle was enhanced in *C. gigas* by high glycogen content in the non-reproductive stage, implying that increased energy metabolism occurs in the high-glycogen Pacific oysters.

**Figure 4:**
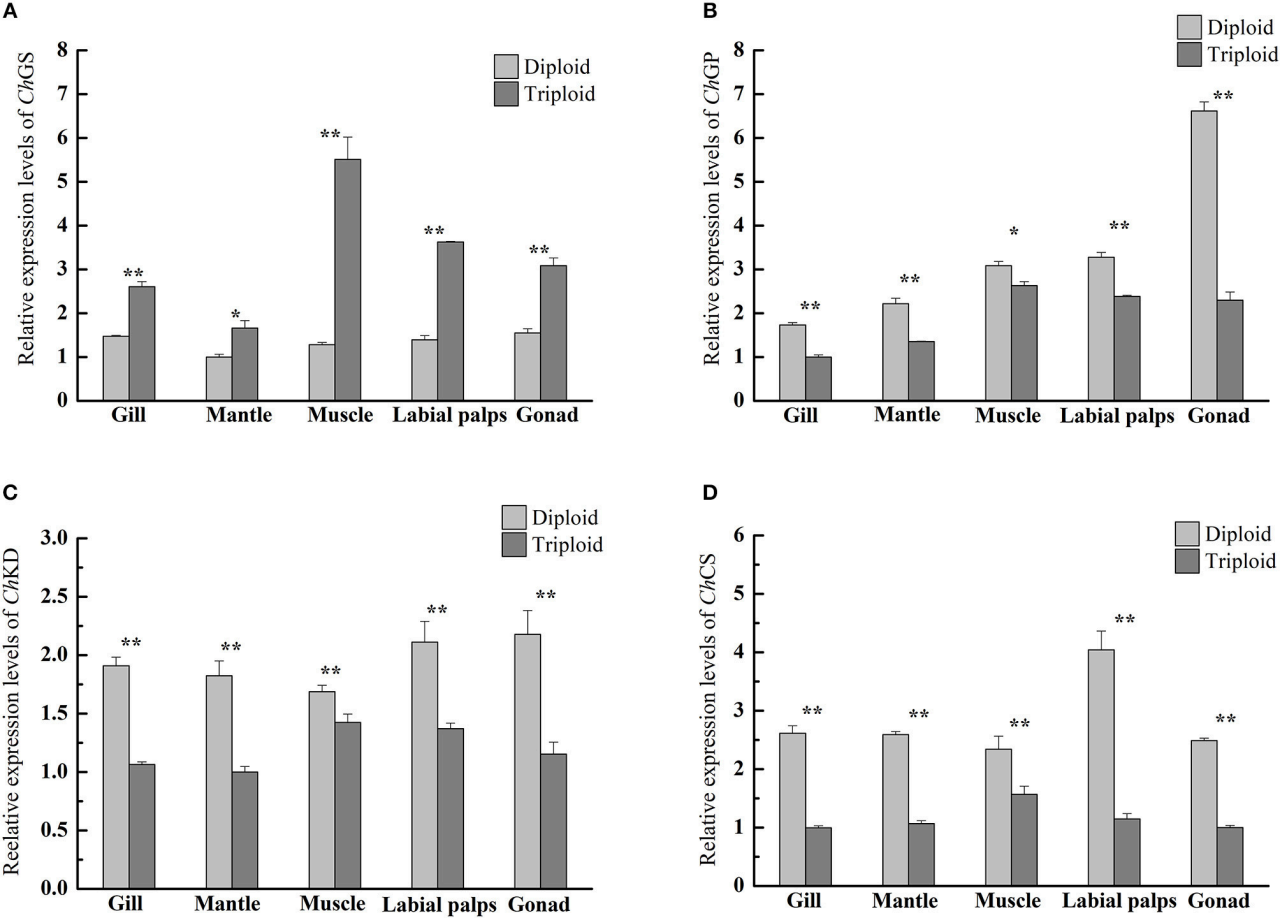
Quantitative PCR analysis of relative mRNA expressions of *Ch*GS **(A)**, *Ch*GP **(B)**, *Ch*KD **(C)**, and *Ch*CS **(D)** in various tissues (gill, mantle, muscle, labial palps, and gonad) of diploid and triploid Hong Kong oysters in the reproductive phase. Each bar represents the mean of the normalized expression levels of the replicates (*n* = 3). Significant differences are indicated by asterisks (^*^ and ^**^ represent *P* < 0.05 and *P* < 0.01, respectively).

In the reproductive phase, the glycogen contents of all five tissues (gill, mantle, muscle, labial palps, and gonad) of triploid Hong Kong oysters were significantly higher than those of diploids, but the protein content of the gonad of diploids was significantly higher than that of triploids. Correspondingly, the relative expressions of *Ch*KD and *Ch*CS in the five tissues of triploids were significantly lower (*P* < 0.05) than in diploids (Figures 4C,D). In addition, from the non-reproductive to the reproductive phase, the glycogen content of the diploid gonad decreased more and the protein content of the diploid gonad increased more than those of triploids. Previous studies on *O. edulis* and *C. gigas* confirmed that glycogen was a preferred energy form for supporting gametogenesis and growth (Pogoda et al., 2013; Li et al., 2017). The simultaneous glycogen decrease and protein increase may indicate the conversion of glycogen into protein during gonad development, as suggested in previous studies on other oyster species such as *C. gigas* and O. e*dulis* (Ruiz et al., 1992; Dridi et al., 2007). Moreover, triploids undergo less gonad development than diploids, and therefore require less energy to support gonad development. This may account for the variations in biochemical composition and relative expressions of *Ch*KD and *Ch*CS of diploid and triploid Hong Kong oysters.

## Author contributions

YQ, ZY, and YZ designed experiments. YQ carried all of the experiments with the help of HM, XW, SX, JL, and RM. YQ analyzed the data and wrote the paper. ZY and YZ critically revised the manuscript and approved the final version to be published.

### Conflict of interest statement

The authors declare that the research was conducted in the absence of any commercial or financial relationships that could be construed as a potential conflict of interest.
